# Diagnostic Accuracy of the LiverRisk Score to Detect Increased Liver Stiffness Among a United States General Population and Subgroups^☆^

**DOI:** 10.1016/j.jceh.2025.102512

**Published:** 2025-02-06

**Authors:** Laurens A. van Kleef, Jesse Pustjens, Harry L.A. Janssen, Willem P. Brouwer

**Affiliations:** ∗ Department of Gastroenterology and Hepatology, Erasmus MC, University Medical Center, Rotterdam, the Netherlands; † Toronto Centre for Liver Disease, Toronto General Hospital, University Health Network, Canada

**Keywords:** external validation, LiverRisk score, MASLD, epidemiology, fibrosis

## Abstract

**Background:**

The LiverRisk score (LRS) has recently been proposed to predict liver fibrosis and future development of liver-related outcomes in the general population. Here, we performed an external validation of this score.

**Methods:**

We used data from National Health and Nutrition Examination Survey 2017-2020, a United States population-based cohort to assess the diagnostic accuracy of the LRS to detect a liver stiffness measurement (LSM) ≥8 and ≥12 kPa. Performance was tested among the entire general population and clinically relevant subgroups.

**Results:**

The cohort comprised 7,025 participants (aged 49 [33-63], 49% male), and 9.7% had an LSM ≥8 and 3.2% had an LSM ≥12 kPa. The area under the receiver characteristic operator curve (AUC) in the overall population was 0.73 (95% confidence interval [CI] :0.71-0.75) and 0.78 (95% CI: 0.74-0.81) to detect an LSM ≥8 and ≥ 12 kPa, respectively, significantly outperforming the fibrosis 4 index (FIB-4) but not the nonalcoholic fatty liver disease fibrosis score, steatosis-associated fibrosis estimator (SAFE), or metabolic dysfunction–associated fibrosis 5 (MAF-5). Performance was consistent among most subgroups, but AUC levels to detect an LSM ≥8 kPa decreased to <0.70 among participants aged 18-40 or 60-80 years, blacks, and individuals with diabetes or liver steatosis. The LRS categorized 80.5% as very low risk, 17.7% as low risk, and 1.8% as at risk, prevalence of an LSM ≥8 in these groups was 6.3%, 20.8%, and 50.5%, respectively. The sensitivity to detect an LSM ≥8 kPa was 47.3% in the overall population (but dropped to 21.3% for individuals aged 18-40 years) despite applying the lowest cut-off, which should yield the highest sensitivity.

**Conclusion:**

The LRS score is a promising new tool to predict liver fibrosis; however, its diagnostic accuracy attenuates especially among patients aged 18-40 or 60-80 years. The overall sensitivity was only 47.3% at the lowest LRS cut-off. Further studies assessing cost-benefit ratios according to the LRS compared to FIB-4 and other risk scores such as MAF-5 and SAFE are required to determine its usefulness in referral strategies.

## Graphical abstract

**Figure:**
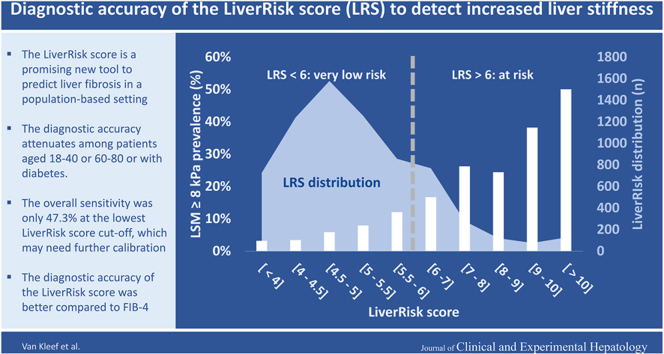

**What is already known on this topic**

Detection of advanced liver disease in low-prevalence populations remains challenging. The LiverRisk score, a noninvasive test to predict fibrosis and long-term complications, has recently been published to aid in this subject.

**What this study adds**

In this external validation among 7,025 participants, the AUC of the LiverRisk score to detect an LSM ≥8 and ≥12 kPa was 0.73 and 0.78, respectively, significantly outperforming the fibrosis 4 index (FIB-4) but not the nonalcoholic fatty liver disease fibrosis score (NFS), steatosis-associated fibrosis estimator (SAFE), or metabolic dysfunction–associated fibrosis 5 (MAF-5). The sensitivity at the low-risk cut-off to detect an LSM ≥8 kPa was 47.3%. Performance attenuated in individuals aged 18-40 or 60-80 years and with diabetes or steatosis.

**How this study might affect research, practice, or policy**

The LiverRisk score can be used for risk stratification. However, it requires careful interpretation since it may not rule out advanced liver disease, especially among participants aged 18-40 or 60-80 years or with profound metabolic dysfunction.

Steatotic liver disease (SLD) has an estimated prevalence of over 30% and has thereby become the most prevalent chronic liver disease.^1^ It is expected that it will replace viral hepatitis in the coming decade as the leading cause of advanced liver disease.^2^ Fortunately, most patients with SLD do not have any symptoms and will not develop advanced liver disease.^3^, ^4^, ^5^ However, this illustrates the challenge to detect those who will eventually develop decompensated liver disease and may have benefited from early referral, lifestyle changes, and medical treatment.^6^ Despite several noninvasive tests being available, it remains challenging to identify those who have liver fibrosis and are therefore at risk of liver-related events like decompensated cirrhosis and hepatocellular carcinoma (HCC).

Recently, the LiverRisk score (LRS) has been published by the LiverScreen consortium, which aims to identify individuals at risk for future liver-related outcomes.^7^ The LRS was developed in a general population and included age, sex, glucose, cholesterol, aspartate aminotransferase (AST), alanine aminotransferase (ALT), gamma-glutamyl transferase (GGT), and platelets and was calibrated to detect increased liver stiffness. In their derivation and validation cohorts, the area under the receiver characteristic operator curve (AUC) to detect a liver stiffness ≥10 kPa was 0.88 and 0.77–0.83, respectively. It significantly outperformed other biomarkers of fibrosis. Importantly, in the prognostication phase, the LRS was associated with a higher risk for liver-related complications and mortality in the UK Biobank cohort.

The LRS may fill an urgent need to identify participants with increased liver stiffness who may opt for hepatic health assessment and hepatologist referral. Although performance across subgroups regarding liver-related events was performed, no data were presented on its ability to detect increased liver stiffness across clinically relevant subgroups. Moreover, despite previously reported concerns about including age in noninvasive scores, the LRS includes age, which may impact the performance across age subgroups.^8^, ^9^, ^10^

Hence, in this study, we want to externally validate the LRS regarding its ability to detect increased liver stiffness across clinically relevant subgroups in a United States, multiethnic general population by using the 2017-2020 National Health and Nutrition Examination Survey (NHANES) cohort.

## METHODS

### Ethical Consent

Participants of the NHANES 2017-2020 cycle provided informed consent. This study was conducted according to the principles as outlined in the Declaration of Helsinki. Data collection for NHANES has been approved by the National Center for Health Statistics Research Ethics Review Board.

### Study Population

For the purpose of this study, NHANES 2017-2020 data were used. Briefly, the NHANES was designed to assess individuals’ health and nutritional status throughout the United States. Data were collected by extensive interviews, physical examination, clinical measurements, and tests by trained research assistants. These tests included laboratory measurements and transient elastography, which allows for the calculation of the LRS and validation against liver stiffness measurement (LSM). Further details on the aims, procedures, and design are available elsewhere.^11^^,^^12^ For the specific purpose of this study, we excluded participants with incomplete data on LRS components as well as patients with potentially unreliable LSM due to the presence of heart failure.^13^ Data are publicly available from the NHANES database. (https://www.cdc.gov/nchs/nhanes/index.htm).

### Biomarker-based Noninvasive Scores

Since the formula of the LRS was not publicly available, it was calculated online using their dedicated tool available at www.liverriskscore.com on the 5th of January 2024. We calculated the fibrosis-4 index (FIB-4), nonalcoholic fatty liver disease fibrosis score (NFS), steatosis-associated fibrosis estimator (SAFE) and metabolic dysfunction–associated fibrosis 5 (MAF-5) following the formulas mentioned afterward.^14^, ^15^, ^16^, ^17^

SAFE=2.97∗Age+154.85∗ln(AST)−58.23∗ln(ALT)+195.48∗ln(globulin)−141.61∗ln(platelets)+62.85IFdiabetes−75

$$NFS=-1.675+0.037*Age+0.094*BMI+1.13\;IF\;\left(pre\right)diabetes+0.99*\;\frac{AST}{ALT}-0.013*platelets-0.66*albumin$$

$$FIB4=\frac{Age*\;AST}{platelets*\;\sqrt{ALT}}$$

MAF-5=−11.3674+waistcircumference∗0.0282−BMI∗0.1761+waistcircumference∗BMI∗0.0019+2.0762fordiabetes+ln(AST)∗2.9207−platelets∗0.0059

Age was expressed in years, body mass index (BMI) in kg/m^2^, waist circumference in cm, AST and ALT in U/L, platelets as 10^9^/L, and albumin and globulin in g/dL.

### Transient Elastography

Participants underwent transient elastography after a minimal 3-h fast using the FibroScan model 502 V2 Touch equipped with an M and XL probe (FibroScan, Echosens, Paris) to assess liver stiffness. Measurements were considered valid if at least 10 measurements were obtained with an interquartile range <30%. Fibrosis was defined as a liver stiffness ≥8 kPa.^18^ Steatosis was assessed by a same-session controlled attenuation parameter (CAP) measurement. CAP levels ≥275 dB/m were used to diagnose steatosis.^6^

### Covariates

Research assistants systematically collected data among all participants, including age, race, and anthropometrics (length, height, and waist circumference). Questionnaires included questions on the presence of heart failure. Blood samples were taken and analyzed for among others ALT, AST, albumin, globulin, platelets, triglycerides, and high-density lipoprotein cholesterol.

Metabolic dysfunction was defined according to the set of criteria provided for the definition of MASLD and included the presence of at least one of the following criteria: overweight, (pre)diabetes, hypertension, high waist circumference, or dyslipidemia.^19^

### Statistical Analysis

First, we performed AUC analysis for the detection of an LSM ≥8 kPa and an LSM ≥12 kPa. The performance of the LRS was compared to the performance of the FIB-4, MAF-5, NFS, and SAFE in this general population cohort. AUCs were assessed for significant differences using the DeLong's test. Next, we assessed the increased LSM distribution for LRS categories. In addition, we calculated the diagnostic accuracy (sensitivity, specificity, negative predictive value [NPV], and positive predictive value [PPV]) of the LRS at the prespecified cut-offs for very low risk and low risk (6 and 10) in the detection of an LSM ≥8 and ≥12 kPa. Performance was tested firstly among the entire general population and secondly among clinically relevant subgroups. These subgroups include participants aged 18-40, 40-60, and 60-80 years, sex (male/female), race (White, Black, Hispanic, or Asian), steatosis (yes/no) diabetes (yes/no), dyslipidaemia (yes/no), BMI (<30 kg/m^2^/≥ 30 kg/m^2^), and metabolic dysfunction (yes/no). Furthermore, we visualized the fibrosis risk as assessed by the LRS against the actual LSM prevalence of ≥8 kPa. Analyses were performed in R (version 4.0.4; Foundation for Statistical Computing, Vienna, Austria). *P* values <0.05 were considered statistically significant.

## RESULTS

### Participants

The cohort comprised 7,768 participants with LSM data. We excluded 186 participants for the presence of heart failure and 557 participants for lack of data on the LRS components, leaving 7,025 participants for analysis. The median age was 49 (33-63) years, and 49% were male. Among them, 9.7% had an LSM ≥8 and 3.2% had an LSM ≥12 kPa. The LRS predefined cut-offs were <6 for very low risk, 6-10 for low risk, 10-15 for medium risk, and >15 for high risk of liver-related events and were present in 80.5%, 17.7%, 1.5%, and 0.3% of the included population, respectively; prevalence of an LSM ≥8 kPa in these risk groups was 6.3%, 20.8%, 47.7%, and 63.2%, respectively. Specific details about these subgroups are available in Table 1. Due to the limited numbers of medium- and high-risk participants in this general population, these have been combined in this paper.

**Table 1:** Baseline Characteristics Stratified for LRS Category.

	LRS: <6	LRS: 6-10	LRS: 10-15	LRS: ≥15
n	5656	1243	107	19
**Demographics**				
Age (years)	45 [31, 60]	62 [52, 72]	57 [47, 62]	54 [50.50, 64]
Male	2468 (43.6)	900 (72.4)	65 (60.7)	15 (78.9)
Race				
White	1943 (34.4)	444 (35.7)	23 (21.5)	5 (26.3)
Black	1403 (24.8)	299 (24.1)	34 (31.8)	5 (26.3)
Hispanic	1312 (23.2)	298 (24.0)	36 (33.6)	7 (36.8)
Asian	708 (12.5)	139 (11.2)	9 (8.4)	2 (10.5)
Other	290 (5.1)	63 (5.1)	5 (4.7)	0 (0.0)
Current smoking	961 (17.0)	214 (17.2)	27 (25.2)	7 (36.8)
**Metabolic health**				
BMI (kg/m^2^)	29.3 (7.2)	30.7 (6.8)	32.2 (7.6)	25.8 (6.3)
Diabetes	509 (9.0)	637 (51.2)	84 (78.5)	9 (47.4)
Dyslipidaemia	2754 (48.7)	897 (72.2)	86 (80.4)	13 (68.4)
**Biochemistry**				
AST (U/L)	19 [16, 22]	23 [18, 31]	30 [17, 51]	107 [32, 189]
ALT (U/L)	16 [12, 23]	24 [17, 38]	36 [22, 61]	108 [54, 151]
HDL-C (mmol/L)	1.4 (0.4)	1.3 (0.4)	1.2 (0.4)	1.8 (1.0)
Triglycerides (mmol/L)	1.2 [0.9, 1.8]	1.4 [1.0, 2.1]	1.9 [1.3, 3.1]	1.6 [1.1, 3.1]
Platelets (10^9^/L)	255 (63)	216 (61)	207 (60)	198 (60)
**Liver stiffness (kPa)**				
Continuous	4.8 [4.0, 5.8]	5.9 [4.7, 7.5]	7.6 [5.1, 11.3]	9.7 [6.1, 14.2]
≥8 kPa	359 (6.3)	259 (20.8)	51 (47.7)	12 (63.2)
≥12 kPa	89 (1.6)	101 (8.1)	24 (22.4)	8 (42.1)

Data is presented as mean (SD), median, [P25-P75] or n and percentage.

ALT, alanine aminotransferase; AST, aspartate aminotransferase; BMI, body mass index; LRS, LiverRisk score; HDL-C, high-density lipoprotein cholesterol.

### LRS Performance in the Overall Population

Among the overall population, the LRS showed an AUC of 0.726 (95% confidence interval [CI]: 0.705-0.747) in the detection of an LSM ≥8 kPa and an AUC of 0.775 (95% CI: 0.742-0.807) in the detection of an LSM ≥12 kPa. When compared to the FIB-4, NFS, and SAFE score; the LRS only outperformed the FIB-4 (*P* < 0.001) and not the NFS and SAFE. In fact, the SAFE score outperformed the LRS (*P* = 0.008) in the detection of an LSM ≥8 kPa. Similar results were obtained for the detection of an LSM ≥12 kPa. As previously shown, the MAF-5 outperformed all of the other aforementioned scores both for LSMs ≥8 and ≥12 kPa.^17^

**Figure 1:**
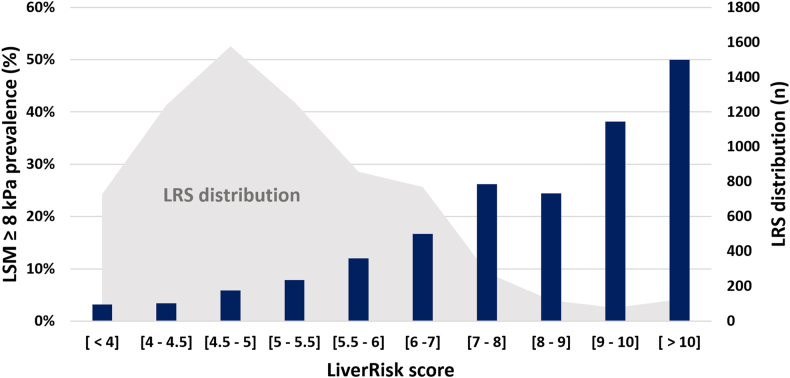
Prevalence of a liver stiffness ≥ 8 kPa per LiverRisk score category together with the LiverRisk score distribution among the general population. LRS distribution among the general population and risk of an LSM ≥8 kPa. The population comprised 7,025 individuals of which 9.7% had an LSM ≥8 kPa. Abbreviations: LRS, liver risk score; LSM, liver stiffness measurement. Data are presented as mean (SD), median, [P25-P75], or n and percentage; ALT, alanine aminotransferase; AST, aspartate aminotransferase; BMI, body mass index; HDL-C, high-density lipoprotein cholesterol; P25-P75, 25th to 75th percentile; SD, standard deviation.

Among the 1,369 (19.5%) participants with an LRS ≥6, the prevalence of an LSM ≥8 kPa was 23.5% and that of an LSM ≥12 kPa was 9.7%. Among those with an LRS ≥10 (n = 126, [1.8%]), the prevalence increased to 50.0% and 25.4%. A higher LRS was associated with a higher fibrosis prevalence (Figure 1).

### LRS Performance Across Subgroups

The AUC to detect an LSM ≥8 kPa was consistent across most subgroups; however, it dropped to below 0.700 for participants aged <40 or ≥60 years, Black ethnicity and individuals with steatosis BMI ≥30 kg/m^2^, or diabetes. Although the AUCs for the detection of an LSM ≥12 kPa were higher, similar drops in performance were observed in these aforementioned subgroups compared to the overall population (Table 2).

**Table 2:** AUC for LRS to Detect Increased Liver Stiffness for the Entire Population and Subgroups.

	LSM ≥ 8 kPa		LSM ≥ 12 kPa	
	AUC	95% CI	AUC	95% CI
**Entire population**	0.726	0.705–0.747	0.775	0.742–0.807
**Age subgroups**				
18–40	0.685	0.634–0.736	0.728	0.643–0.812
40–60	0.731	0.695–0.767	0.789	0.730–0.848
60–80	0.681	0.648–0.714	0.731	0.682–0.779
**Race**				
White	0.738	0.705–0.772	0.745	0.691–0.798
Black	0.659	0.614–0.703	0.725	0.643–0.806
Hispanic	0.729	0.687–0.772	0.799	0.734–0.864
Asian	0.805	0.747–0.863	0.884	0.827–0.941
**Sex**				
Male	0.708	0.679–0.736	0.745	0.698–0.791
Female	0.735	0.703–0.767	0.803	0.757–0.850
**Steatosis**				
Yes	0.673	0.647–0.700	0.700	0.658–0.743
No	0.730	0.690–0.771	0.837	0.779–0.896
**Diabetes mellitus**				
Yes	0.612	0.574–0.651	0.762	0.729–0.796
No	0.698	0.670–0.726	0.753	0.705–0.801
**Dyslipidaemia**				
Yes	0.702	0.676–0.728	0.738	0.699–0.778
No	0.728	0.690–0.767	0.798	0.736–0.860
**Metabolic dysfunction**				
Yes	0.713	0.691–0.735	0.762	0.729–0.796
No	0.861	0.786–0.935	0.957	0.888–1.000
**BMI**				
≥30 kg/m^2^	0.684	0.657–0.711	0.710	0.668–0.752
<30 kg/m^2^	0.786	0.752–0.819	0.886	0.848–0.923

AUC is calculated for the entire population (n = 7,025) and clinically relevant subgroups for the detection of LSMs ≥8 kPa and ≥12 kPa.

Abbreviations: AUC, area under the receiver characteristic operator curve; BMI, body mass index; CI, confidence interval; LRS, LiverRisk score; LSM, liver stiffness measurement.

### LRS Performance at Different Cut-offs

Details on the prevalence of LSMs ≥8 and ≥12 kPa based on LRS outcomes are depicted in Table 3. In general, 80.5% of participants were considered to be at very low risk for liver-related events (LRS < 6); however, despite being considered very low risk, 6.3% still had an LSM ≥8 kPa. This increased to 17.5% in participants with diabetes. As previously outlined, most participants classify as very low risk (80.5%), but this was even higher among participants aged 18-40 (94.8%) and females (89.1%), while in these groups, the majority of liver fibrosis cases were found. In fact, 79% of cases with an LSM ≥8 kPa among participants aged 18-40 years and 63% of cases with an of LSM ≥8 kPa among females remained undetected with the lowest LRS cut-off.

**Table 3:** LSM Distribution Stratified for LRS for the Overall Population and Subgroups.

Population		LRS	distribution	LSM < 8 kPa	LSM ≥ 8 kPa	LSM ≥ 12 kPa
**Overall**		<6	80.5%	93.7%	6.3%	1.6%
		6–10	17.7%	79.2%	20.8%	8.1%
		>10	1.8%	50.0%	50.0%	25.4%
**Age**	**18–40**	<6	94.8%	95.3%	4.7%	1.2%
		6–10	4.6%	80.4%	19.6%	8.9%
		>10	0.6%	53.3%	46.7%	33.3%
	**40–60**	<6	79.8%	93.7%	6.3%	1.4%
		6–10	17.4%	78.5%	21.5%	7.5%
		>10	2.7%	54.0%	46.0%	20.6%
	**60**–**80**	<6	66.2%	91.1%	8.9%	2.4%
		6–10	31.7%	79.3%	20.7%	8.3%
		>10	2.1%	43.8%	56.3%	29.2%
**Race**	**White**	<6	80.5%	93.9%	6.1%	2.0%
		6–10	18.4%	76.1%	23.9%	9.7%
		>10	1.2%	50.0%	50.0%	25.0%
	**Black**	<6	80.6%	91.9%	8.1%	1.6%
		6–10	17.2%	82.6%	17.4%	4.7%
		>10	2.2%	53.8%	46.2%	20.5%
	**Hispanic**	<6	79.4%	93.4%	6.6%	1.4%
		6–10	18.0%	80.5%	19.5%	8.7%
		>10	2.6%	46.5%	53.5%	30.2%
	**Asian**	<6	82.5%	96.5%	3.5%	0.7%
		6–10	16.2%	81.3%	18.7%	6.5%
		>10	1.3%	63.6%	36.4%	18.2%
**Sex**	**Male**	<6	71.6%	92.9%	7.1%	1.9%
		6–10	26.1%	80.9%	19.1%	7.2%
		>10	2.3%	46.3%	53.8%	25.0%
	**Female**	<6	89.1%	94.2%	5.8%	1.3%
		6–10	9.6%	74.6%	25.4%	10.5%
		>10	1.3%	56.5%	43.5%	26.1%
**Steatosis**	**Yes**	<6	70.9%	87.9%	12.1%	3.3%
		6–10	26.3%	74.5%	25.5%	9.6%
		>10	2.8%	46.3%	53.7%	25.6%
	**No**	<6	87.4%	97.0%	3.0%	0.6%
		6–10	11.6%	86.7%	13.3%	5.7%
		>10	1.1%	56.8%	43.2%	25.0%
**Diabetes mellitus**	**Yes**	<6	41.1%	82.5%	17.5%	4.9%
		6–10	51.4%	76.9%	23.1%	7.8%
		>10	7.5%	52.7%	47.3%	21.5%
	**No**	<6	89.0%	94.8%	5.2%	1.2%
		6–10	10.5%	81.5%	18.5%	8.4%
		>10	0.6%	42.4%	57.6%	36.4%
**Dyslipidaemia**	**Yes**	<6	73.4%	91.7%	8.3%	2.2%
		6–10	23.9%	78.8%	21.2%	7.8%
		>10	2.6%	51.5%	48.5%	21.2%
	**No**	<6	88.6%	95.5%	4.5%	1.0%
		6–10	10.6%	80.1%	19.9%	9.0%
		>10	0.8%	44.4%	55.6%	40.7%
**Metabolic dysfunction**	**Yes**	<6	78.9%	93.1%	6.9%	1.7%
		6–10	19.2%	79.3%	20.7%	8.2%
		>10	1.9%	49.6%	50.4%	25.6%
	**No**	<6	95.8%	97.8%	2.2%	0.2%
		6–10	3.4%	72.7%	27.3%	4.5%
		>10	0.8%	60.0%	40.0%	20.0%
**BMI**	**≥30 kg/m**^**2**^	<6	76.6%	87.8%	12.2%	3.4%
		6–10	21.2%	72.0%	28.0%	11.0%
		>10	2.2%	43.5%	56.5%	27.4%
	**<30 kg/m**^**2**^	<6	83.4%	97.3%	2.7%	0.4%
		6–10	15.1%	85.9%	14.1%	5.4%
		>10	1.4%	60.0%	40.0%	20.0%

LRS cut–offs were based on predefined liver–related events risk categories, in which an LRS <6 indicates very low risk, LRS 6–10 indicates low risk, and LRS >10 indicates medium and high risk.

Abbreviations: BMI, body mass index; LRS, liver risk score; LSM, liver stiffness measurement.

### Diagnostic Parameters of the LRS

Sensitivity, specificity, NPV, and PPV for the detection of an increased LSM are provided for LRS 6 (Table 4) and LRS 10 (Table 5). The sensitivity of the LRS to detect an LSM ≥12 kPa was better than that for detecting an LSM ≥8 kPa at both investigated LRS thresholds. What stands out is the poor sensitivity of the LRS, even at its most lenient cut-off (LRS: 6), to detect an LSM ≥8 kPa and an LSM ≥12 kPa (21.3% and 35.7%, respectively) among individuals aged 18-40 years. Other subgroups in which sensitivity is substantially poorer than the overall population are females and participants without diabetes. As a result of the poor sensitivity, the NPV (in light of the background prevalence) is low across all subgroups.

**Table 4:** Diagnostic Accuracy of the LRS at the Threshold for Very Low Risk (LRS = 6) in the Detection of Increased LSM in the General Population and Clinically Relevant Subgroups.

	prev	Detection of an LSM ≥8 kPa				prev	Detection of an LSM ≥12 kPa			
		sens	spec	PPV	NPV		sens	spec	PPV	NPV
**All**	9.7%	47.3%	83.5%	23.5%	93.7%	3.2%	59.9%	81.8%	9.7%	98.4%
**Age groups**										
18-40	5.6%	21.3%	95.7%	22.8%	95.3%	1.7%	35.7%	95.3%	11.8%	98.8%
40-60	10.1%	49.8%	83.1%	24.8%	93.7%	3.0%	63.2%	81.1%	9.3%	98.6%
60-80	13.6%	56.7%	69.8%	22.8%	91.1%	4.9%	67.0%	67.9%	9.6%	97.6%
**Race**										
White	9.9%	50.2%	83.8%	25.4%	93.9%	3.6%	56.8%	81.9%	10.6%	98.0%
Black	10.5%	38.3%	82.8%	20.7%	91.9%	2.6%	48.9%	81.4%	6.5%	98.4%
Hispanic	10.1%	48.5%	82.5%	23.8%	93.4%	3.5%	67.2%	81.1%	11.4%	98.6%
Asian	6.4%	54.5%	85.1%	20.0%	96.5%	1.9%	68.8%	83.5%	7.3%	99.3%
**Sex**										
Male	11.3%	55.1%	75.0%	21.9%	92.9%	3.8%	64.9%	73.0%	8.7%	98.1%
Female	8.1%	36.8%	91.4%	27.5%	94.2%	2.5%	52.7%	90.2%	12.3%	98.7%
**Steatosis**										
Yes	16.8%	48.9%	74.9%	28.2%	87.9%	5.6%	58.3%	72.6%	11.2%	96.7%
No	4.6%	43.2%	88.9%	15.8%	97.0%	1.4%	64.4%	88.1%	7.3%	99.4%
**Diabetes**										
Yes	22.6%	68.2%	43.8%	26.2%	82.5%	7.7%	73.7%	42.3%	9.6%	95.1%
No	6.9%	32.7%	90.6%	20.5%	94.8%	2.2%	49.6%	89.8%	9.9%	98.8%
**Dyslipidaemia**										
Yes	12.5%	51.0%	76.9%	23.9%	91.7%	4.1%	59.9%	74.8%	9.1%	97.8%
No	6.5%	39.3%	90.6%	22.5%	95.5%	2.1%	60.0%	89.7%	11.3%	99.0%
**Metabolic dysfunction**										
Yes	10.3%	47.6%	82.0%	23.4%	93.1%	3.4%	59.8%	80.3%	9.8%	98.3%
No	3.4%	36.4%	97.0%	29.6%	97.8%	0.5%	66.7%	96.1%	7.4%	99.8%
**BMI**										
≥30 kg/m^2^	16.6%	43.5%	80.5%	30.7%	87.8%	5.6%	52.9%	78.3%	12.6%	96.6%
<30 kg/m^2^	4.9%	55.1%	85.4%	16.4%	97.3%	1.5%	75.4%	84.3%	6.7%	99.6%

The total population comprised 7,025 participants of which 9.7% had an LSM ≥8 kPa and 3.2% had an LSM ≥12 kPa.

Abbreviations: BMI, body mass index; LRS, liver risk score; LSM, liver stiffness measurement; NPV, negative predictive value; prev, prevalence; PPV, positive predictive value; sens, sensitivity; spec, specificity.

**Table 5:** Diagnostic Accuracy of the LRS at the Threshold for Medium Risk (LRS = 10) in the Detection of Increased LSM in the General Population and Clinically Relevant Subgroups.

	prev	Detection of an LSM ≥8 kPa				prev	Detection of an LSM ≥12 kPa			
		sens	spec	PPV	NPV		sens	spec	PPV	NPV
**All**	9.7%	9.3%	99.0%	50.0%	91.0%	3.2%	14.4%	98.6%	25.4%	97.2%
**Age groups**										
18-40	5.6%	5.1%	99.7%	46.7%	94.6%	1.7%	11.9%	99.6%	33.3%	98.5%
40-60	10.1%	12.6%	98.4%	46.0%	91.0%	3.0%	19.1%	97.8%	20.6%	97.5%
60-80	13.6%	8.6%	98.9%	56.3%	87.3%	4.9%	12.5%	98.4%	29.2%	95.7%
**Race**										
White	9.9%	5.9%	99.4%	50.0%	90.6%	3.6%	8.0%	99.1%	25.0%	96.6%
Black	10.5%	9.8%	98.7%	46.2%	90.3%	2.6%	17.8%	98.2%	20.5%	97.8%
Hispanic	10.1%	13.8%	98.7%	53.5%	91.1%	3.5%	22.4%	98.1%	30.2%	97.2%
Asian	6.4%	7.3%	99.1%	36.4%	94.0%	1.9%	12.5%	98.9%	18.2%	98.3%
**Sex**										
Male	11.3%	11.0%	98.8%	53.8%	89.7%	3.8%	15.3%	98.2%	25.0%	96.7%
Female	8.1%	6.9%	99.2%	43.5%	92.3%	2.5%	13.2%	99.0%	26.1%	97.8%
**Steatosis**										
Yes	16.8%	9.0%	98.4%	53.7%	84.3%	5.6%	12.9%	97.8%	25.6%	95.0%
No	4.6%	10.0%	99.4%	43.2%	95.8%	1.4%	18.6%	99.2%	25.0%	98.8%
**Diabetes**										
Yes	22.6%	15.7%	94.9%	47.3%	79.4%	7.7%	21.1%	93.6%	21.5%	93.5%
No	6.9%	4.7%	99.7%	57.6%	93.4%	2.2%	9.4%	99.6%	36.4%	98.0%
**Dyslipidaemia**										
Yes	12.5%	10.3%	98.4%	48.5%	88.5%	4.1%	13.8%	97.8%	21.2%	96.4%
No	6.5%	7.0%	99.6%	55.6%	93.9%	2.1%	15.7%	99.5%	40.7%	98.2%
**Metabolic dysfunction**										
Yes	10.3%	9.3%	99.0%	50.4%	90.4%	3.4%	14.2%	98.5%	25.6%	97.0%
No	3.4%	9.1%	99.5%	40.0%	96.9%	0.5%	33.3%	99.4%	20.0%	99.7%
**BMI**										
≥30 kg/m^2^	16.6%	7.5%	98.9%	56.5%	84.3%	5.6%	10.8%	98.3%	27.4%	94.9%
<30 kg/m^2^	4.9%	11.7%	99.1%	40.0%	95.6%	1.5%	19.7%	98.8%	20.0%	98.8%

The total population comprised 7,025 participants of which 9.7% had an LSM ≥8 kPa and 3.2% had an LSM ≥12 kPa.

Abbreviations: BMI, body mass index; LRS, liver risk score; LSM, liver stiffness measurement; NPV, negative predictive value; prev, prevalence; PPV, positive predictive value; sens, sensitivity; spec, specificity.

## DISCUSSION

We investigated the diagnostic performance and clinical utility of the LRS in the detection of participants with increased liver stiffness defined as LSMs ≥8 kPa and ≥12 kPa. The LRS outperformed the FIB-4 and has an acceptable discriminative value, especially among middle-aged populations (aged 40-60 years). Its performance was independent of the presence of any form of metabolic dysfunction (e.g., hypertension, a BMI ≥25, or dyslipidemia), but its AUC attenuated in younger and older populations and Black individuals, as well as among those with more profound metabolic dysfunction such as diabetes or steatosis. The latter observation may be a significant hurdle as European Association for the Study of the Liver, American Association for the Study of Liver Diseases, and American Gastroenterological Association clinical practice guidelines all recommend screening for liver fibrosis in this population.^6^^,^^20^, ^21^, ^22^

An important strength of the LRS is its development in a predominantly general population.^7^ This corresponds with the population for which noninvasive tests are most urgently needed.^23^ By design, this score is thus calibrated on liver stiffness (and extensively validated) and not primarily calibrated on liver histology data. This prevents the issue with other scores that are calibrated in highly selected patient populations that underwent liver biopsy. These populations reflect only the tip of the iceberg, which is likely not homogeneous to the undetected population (due to selection bias), as illustrated by the poorer performance of noninvasive scores in the general population compared to hospital populations.^24^, ^25^, ^26^, ^27^

Consistent performance of noninvasive tests across subgroups is important for its clinical utility. However, unfortunately, we demonstrated that the performance of the LRS was poorer among those aged 18-40 years and 60-80 years than among those aged 40-60 years. In fact, the sensitivity to detect increased liver stiffness at the lowest cut-off (LRS: 6, which should yield the highest sensitivity) among young participants was only 21%; hence 79% remain undetected. This is problematic, given the expected rise in incidence and prevalence of MASLD and metabolic-dysfunction associated steatohepatitis (MASH) among younger populations. As such, these young individuals may face the highest lifetime risk of liver disease and potentially the greatest need for the detection of at-risk liver disease. In the older group, despite 34% of participants having an LRS ≥6, the fibrosis prevalence remained high (9%) among those with an LRS <6, which may falsely reassure healthcare providers in ruling out advanced liver disease.

Age as a complicating factor is not unique to the LRS since similar issues have previously been demonstrated for FIB-4, NFS, and SAFE.^9^^,^^10^ One can even argue that age (especially as a linear factor) should not be included in non-invasive tests (NITs), as fibrosis can occur at all ages and age itself is likely not the explanatory factor, but rather the increased exposure duration to MASLD and/or presence of metabolic dysfunction.^8^, ^9^, ^10^ Indeed, the recently published MAF-5, which did not include age, demonstrated consistent performance across subgroups in the internal and external validation.^17^^,^^28^ Moreover, the components included in NITs might have less discriminative value in an elderly population, where metabolic dysfunction and other diseases are more prevalent, distorting the algorithms. Finally, individuals who already have fibrosis before the age of 40 years might have a different (sub)phenotype, aligning the discovery of two distinct phenotypes recently.^29^ These factors all contribute to the poorer performance of the LRS and other NITs in younger and older populations, which can only be partially prevented by not including age in algorithms.

Metabolic dysfunction is one of the key drivers of fibrogenesis in the general population and is accounted for by glucose and cholesterol levels in the LRS. Although the LRS performance among those with any form of metabolic dysfunction as defined by the MASLD criteria was similar to the overall population, its performance attenuated substantially when focusing on individuals with more profound forms of metabolic dysfunction in the form of diabetes, a BMI ≥30 kg/m^2^, or steatosis. For example, almost 60% of the diabetes population had an LRS ≥6, but, nonetheless, 32% of those with increased liver stiffness were missed, and still 17% of those with an LRS <6 (very low risk) had an LSM ≥8 kPa. Although less distinct, similar results were found for participants with steatosis. Additionally, the NPV was only slightly better than that of a nondiscriminative test (which is 100% background prevalence). The LRS therefore may have no place in ruling out increased liver stiffness, especially in patients with profound metabolic dysfunction or already known steatosis. This is a substantial issue since all clinical practice guidelines advocate ruling out fibrosis in this particular population.^6^^,^^20^, ^21^, ^22^ Further studies assessing cost-benefit ratios according to the LRS and other risk scores such as MAF-5 and SAFE are required to assess the usefulness in referral strategies.

The LRS is validated against liver related outcomes, which included liver-related death, liver-related hospital admission, and incident HCC in the UK Biobank.^7^^,^^17^ For example, the risk of liver-related events was exceptionally low among those with an LRS <6. For example, <0.1% developed HCC when having an LRS <6 during the median follow-up of 8 years, this cumulative incident rate increased to 0.1%, 1.0%, and 4.4% for an LRS of 6-10, 10-15, and ≥ 15, respectively. At the first glance, the clinical relevance of those having increased liver stiffness while having a low LRS may thus be limited. However, it needs to be stressed that the progression of one stage of fibrosis takes several years; hence, as a result, these individuals with low LRSs while having increased liver stiffness may be at the risk for liver-related events later on.^3^, ^4^, ^5^ In fact, these individuals may be the target population in which liver-related events can be prevented with lifestyle changes and medical treatment when available to prevent further disease progression and finally the occurrence of (decompensated) cirrhosis or HCC.

### Limitations

The following limitations need to be mentioned. First, in this general population cohort, no liver biopsy data were available due to its invasive nature. Instead, LSM was used similar to the development of the LRS. Following the EASL and AASLD guidelines, we used the LSM ≥ 8 kPa and ≥ 12 kPa as clinically relevant cut-offs, rather than the categories used for LRS.^6^^,^^21^ Second, no follow-up data were available on liver-related events, and further validation of the associations with adverse outcomes could not be verified. Third, although most ethnicities are included in the NHANES, the number of Asian participants is low, and its result may therefore be different. Moreover, although it was validated among different ethnicities, all individuals were living in the USA, and the lifestyle may not correspond with the lifestyle of their genetic origin. Further validation is required.

In conclusion, the LRS is a promising new tool that can aid clinicians in identifying those who are at risk of fibrosis. However, like other scores that include age, its performance attenuated beyond its prime population (aged 40-60 years). Moreover, among young patients, those with diabetes or a diagnosis of steatosis, the LRS was not able to accurately rule out increased liver stiffness even at the lowest cut-off. These limitations should be considered in the interpretation of the LRS on an individual basis.

## CREDIT AUTHORSHIP CONTRIBUTION STATEMENT

Collection of data: LvK and WPB; Study design, data analysis, writing of the manuscript: LvK and WPB; Critical review of the manuscript, writing of the manuscript, approval of final version, and approval of submission: all authors.

## FUNDING

Financial support was provided by the Foundation for Liver and Gastrointestinal Research, Rotterdam, the Netherlands. The funding source did not influence the study design, data collection, analysis, and interpretation of the data or the writing of the report and decision to submit for publication.

## DATA TRANSPARENCY STATEMENT

Data are publicly available from the National Health and Nutrition Examination Survey database (https://www.cdc.gov/nchs/nhanes/index.htm).

## CONSENT FOR PUBLICATION

Not appliable.

## DECLARATION OF COMPETING INTEREST

**HLAJ** received grants from AbbVie, Arbutus, Gilead Sciences, Janssen and Roche, and is a consultant for Arbutus, Arena, Enyo, Gilead Sciences, GlaxoSmithKline (GSK), Janssen, Merck, Roche, Vir Biotechnology Inc. and Viroclinics. **WPB** received speakers fees for Eli Lilly, is part of the advisory board of Novo Nordisk and participates in trials of 89BIO, Boehringer Ingelheim, Novo Nordisk, and Inventiva Pharma. The other authors **LvK** and **JP** had no conflicts of interest with respect to the current work.
